# Description of Endobronchial Ultrasound-Guided Transbronchial Mediastinal Cryobiopsy for Mediastinal Lesions: A Real-World Study

**DOI:** 10.7759/cureus.115521

**Published:** 2026-08-31

**Authors:** Jacobo Echeverri-Hoyos, Jaime A Echeverri Franco, Juanita Trujillo-Angulo, Eduardo Tuta-Quintero

**Affiliations:** 1 Department of General Medicine, Institución Universitaria Visión de las Américas, Pereira, COL; 2 Department of Pulmonology, Clínica de Alta Tecnología Oncólogos del Occidente, Pereira, COL; 3 Department of General Medicine, Universidad del Rosario, Bogotá, COL; 4 Department of Epidemiology and Public Health, Universidad de La Sabana, Chía, COL

**Keywords:** cryobiopsy, endobronchial ultrasound, mediastinal, nodal, transbronchial

## Abstract

Endobronchial ultrasound-guided transbronchial mediastinal cryobiopsy (EBUS-TBNC) enables the acquisition of larger and higher-quality tissue samples than conventional endobronchial ultrasound-guided transbronchial needle aspiration (EBUS-TBNA); however, evidence regarding its use and outcomes in Latin America remains limited. This retrospective observational study describes the clinical and procedural characteristics, the frequency of diagnosis obtained by histopathology, and the safety of patients subjected to EBUS-TBNC between June 2023 and December 2025. A total of 118 patients were included; 50.4% were female, and the mean age was 59.6 ± 17.8 years. Initial diagnosis was the main procedural indication (82.2%), and 79.7% of procedures targeted mediastinal lymph nodes, with station 7 being the most frequently biopsied (71.2%). A definitive histopathological diagnosis was obtained in 92.4% (109/118) of patients. The most common complications were pneumomediastinum (11.9%), bronchospasm (5.9%), and pneumothorax (4.2%), with no hemomediastinum or infectious complications reported. Lymphoma was the most frequent neoplasm (27.9%), whereas anthracosis was the predominant benign diagnosis (14.4%). Molecular testing was feasible, with PD-L1 (programmed death-ligand 1) being the most frequently assessed biomarker (37.3%). These findings suggest that EBUS-TBNC is a valuable technique for obtaining tissue suitable for comprehensive histopathological and molecular assessment, supporting its use in the diagnostic evaluation of mediastinal lesions. Its safety profile was characterized predominantly by non-severe complications, supporting its potential role as a diagnostic tool for mediastinal lesions, particularly in clinical scenarios where larger tissue samples are required, such as the evaluation of lymphoma.

## Introduction

Endobronchial ultrasound-guided transbronchial needle aspiration (EBUS-TBNA) is the minimally invasive procedure of choice for diagnostic evaluation and mediastinal staging in patients with intrathoracic lymphadenopathy and suspected pulmonary malignancy [1,2]. Its high sensitivity, low complication rate, and ability to access multiple mediastinal lymph node stations have enabled EBUS-TBNA to replace more invasive surgical procedures, such as mediastinoscopy, in a substantial proportion of cases [3]. Beyond its role in lung cancer staging, EBUS-TBNA is widely used for the diagnosis of granulomatous, infectious, and neoplastic diseases involving the mediastinum [3-6].

Endobronchial ultrasound-guided transbronchial nodal cryobiopsy (EBUS-TBNC) involves the insertion of a cryoprobe through the tract previously created by the EBUS-TBNA needle, allowing the acquisition of larger tissue specimens with better preservation of lymph node architecture [7,8]. Recent studies and meta-analyses have demonstrated that mediastinal cryobiopsy can achieve diagnostic yields exceeding 90% [9], with particular utility in the diagnosis of lymphomas, granulomatous diseases, and poorly differentiated tumors, while maintaining a safety profile comparable to that of conventional EBUS-TBNA [7,8].

Currently, most of the available evidence originates from studies conducted in Europe, Asia, and North America [7,8]. However, it remains unclear whether these findings are directly applicable to Latin American settings, where differences in disease epidemiology, healthcare resources, and clinical practice may influence the use and outcomes of EBUS-TBNC. Data from Latin America remain scarce despite the region’s high burden of granulomatous infectious diseases, such as tuberculosis, and malignant mediastinal diseases [4,5]. Furthermore, access to molecular testing and advanced interventional bronchoscopy technologies varies considerably across Latin American healthcare systems [9-11]. To address this evidence gap, the present study aimed to describe the clinical and procedural characteristics, safety profile, and histopathological findings of patients undergoing EBUS-TBNC at a referral center for respiratory diseases in Latin America.

## Materials and methods

Study design

A retrospective observational study was conducted including consecutive patients who underwent EBUS-TBNC at a referral center for respiratory diseases. This was a retrospective study, and informed consent was not required. The reporting of this study conforms to the STROBE (STrengthening the Reporting of OBservational studies in Epidemiology) guidelines [9].

This study was reviewed and approved by the Research Committee of Oncólogos del Occidente under approval number RG-FO-001. Given the retrospective observational design of the study and the use of de-identified data, the requirement for informed consent was waived by the Ethics Committee. The study was conducted in accordance with the ethical principles of the Declaration of Helsinki and applicable local regulations.

Eligibility criteria

All consecutive adult patients (≥18 years) who underwent EBUS-TBNC between June 2023 and December 2025 for the diagnostic evaluation, staging, or restaging of mediastinal lymph nodes or pulmonary lesions were screened for eligibility. All eligible consecutive patients were included to minimize selection bias, provided that complete clinical, procedural, and histopathological data were available. Patients with incomplete records for the variables of interest or in whom tissue samples could not be successfully obtained using the study technique were excluded.

Variables and definitions

Demographic, clinical, procedural, diagnostic, and safety variables were collected from institutional records. Demographic variables included age and sex. Clinical variables comprised a history of pulmonary or extrapulmonary malignancy, indication for the procedure (initial diagnosis, staging, or restaging), and preoperative physical status according to the American Society of Anesthesiologists (ASA) classification.

Procedural variables included the lymph node station according to the International Association for the Study of Lung Cancer (IASLC) lymph node map, the number of sampled stations, number of needle passes, number of cryobiopsies performed, freezing time, and the size of the sampled lymph node or pulmonary lesion, as these are established procedural parameters for EBUS-guided mediastinal sampling [10]. In addition, ultrasonographic characteristics of mediastinal lymph nodes were recorded, including shape, margin definition, echogenicity, presence of calcifications, central necrosis, and visualization of the hilar structure [11].

Safety variables included complication grading according to the classification proposed by Folch [10], as well as the occurrence of pneumomediastinum, pneumothorax, hemomediastinum, bronchospasm, and infectious complications [10,11]. Final diagnoses, positron emission tomography-computed tomography (PET-CT) findings, and molecular analyses were also recorded, including programmed death-ligand 1 (PD-L1) expression and alterations in epidermal growth factor receptor (EGFR), anaplastic lymphoma kinase (ALK), Kirsten rat sarcoma viral oncogene homolog (KRAS), and B-Raf proto-oncogene serine/threonine kinase (BRAF) [12].

Endobronchial ultrasound-guided procedures

EBUS was performed using a dedicated EBUS bronchoscope equipped with a convex ultrasound transducer at its distal tip, allowing real-time ultrasonographic visualization of mediastinal and hilar lymph nodes adjacent to the tracheobronchial tree [11]. After identification of the target lymph node, the EBUS bronchoscope was positioned to obtain an optimal ultrasound view, and the lymph node was punctured under real-time ultrasound guidance using a dedicated aspiration needle. EBUS-TBNA was performed according to standard techniques.

For CryoEBUS, after identification and puncture of the target lymph node, the needle tract was used as the access route for introduction of a 1.1-mm cryoprobe into the lymph node [10,11]. The cryoprobe was advanced through the puncture site under EBUS guidance and activated to obtain a larger tissue specimen. The probe was subsequently withdrawn together with the frozen specimen, which was immediately retrieved for histopathological evaluation. The procedure was performed by two operators: one operator controlled the EBUS bronchoscope and performed the ultrasound examination and positioning, while the second operator performed the mediastinal puncture and handled the insertion and withdrawal of the cryoprobe.

Representative images of the procedure are shown in Figure 1. Lymphadenopathy at the subcarinal mediastinal station 7, with the 1.1-mm cryoprobe positioned within the lymph node during tissue acquisition, is shown in Figure 1A; the inset demonstrates the endoscopic view of the EBUS bronchoscope positioned at the carina. The cryotherapy console with the cryoprobe connected and the video bronchoscopy tower with the operators performing the procedure using the EBUS bronchoscope, aspiration needle, and cryoprobe are shown in Figure 1B. The two-operator technique is illustrated in Figure 1C, with one operator controlling the bronchoscope and performing EBUS and the second operator performing the mediastinal puncture and managing cryoprobe insertion and withdrawal.

**Figure 1:**
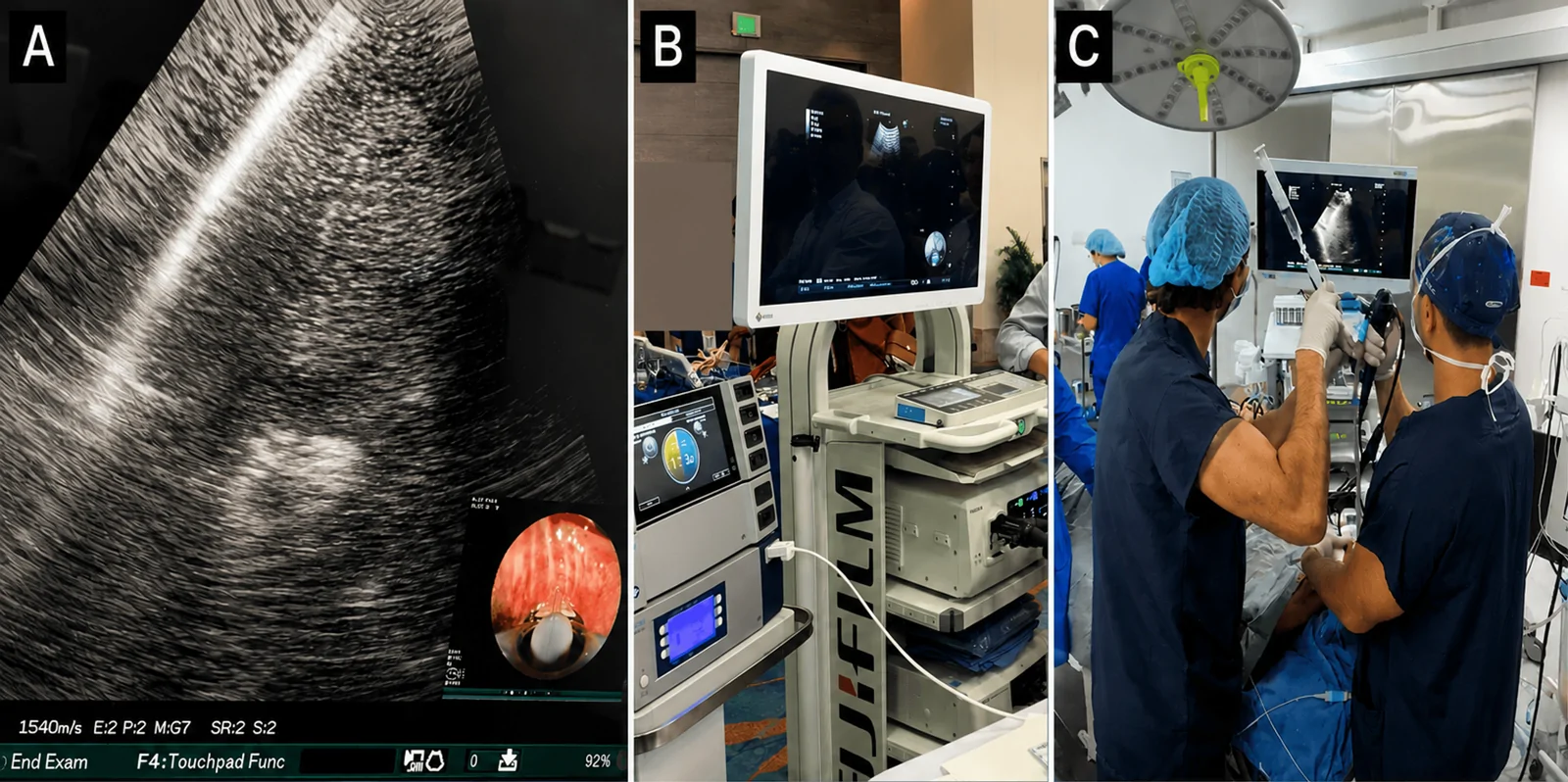
EBUS-guided cryobiopsy of mediastinal lymph nodes: procedural imaging, equipment, and two-operator technique (A) EBUS image demonstrating the target mediastinal lymph node with the cryoprobe positioned within the lesion; the inset shows the corresponding bronchoscopic view during cryobiopsy. (B) EBUS and cryobronchoscopy equipment used for the procedure, including the ultrasound processor and cryotherapy system. (C) Procedural photograph demonstrating the bronchoscopic team performing EBUS-guided transbronchial cryobiopsy under real-time ultrasound guidance. EBUS, endobronchial ultrasound

All procedures were performed by two interventional pulmonologists, each performing more than 100 EBUS procedures annually. The selection of mediastinal lymph node stations or pulmonary lesions for biopsy was based on prior clinical assessment and pre-procedural imaging, including chest computed tomography (CT) and/or PET-CT, when available. Following conventional EBUS-TBNA sampling, transbronchial cryobiopsy specimens were obtained using either a 1.1-mm cryoprobe (approximately 85% of procedures) or a 1.7-mm cryoprobe (approximately 15% of procedures), advanced through the needle tract created during the aspiration procedure. The number of tissue samples obtained and the freezing time used during each procedure were recorded [10,11].

Statistical analysis

Continuous variables were described using means and standard deviations (SD) or medians and interquartile ranges (IQR), according to their distribution. Categorical variables were summarized as absolute frequencies and percentages.

A descriptive analysis of the entire cohort was performed, followed by an exploratory comparison between patients in whom a definitive histopathological or microbiological diagnosis was obtained and those without a definitive diagnosis. Continuous variables were compared using Student's t test or the Mann-Whitney U test, as appropriate, whereas categorical variables were compared using the χ² test or Fisher's exact test [13].

A definitive diagnosis was defined as the establishment of a histopathological or microbiological diagnosis based on specimens obtained by EBUS-TBNC. Benign non-specific findings, including anthracosis and lymphoid hyperplasia, were considered definitive diagnoses only when they were consistent with the clinical, radiological, and follow-up findings documented in the medical records. Safety outcomes were assessed by recording procedure-related complications identified during or after EBUS-TBNC according to the Folch classification [10]. Given the descriptive and exploratory nature of the study, no formal evaluation of diagnostic accuracy or diagnostic performance was performed. A two-sided p-value of <0.05 was considered statistically significant. All statistical analyses were performed using IBM SPSS Statistics version 25 (IBM Corp., Armonk, NY, USA).

## Results

Among the 118 included patients, 50.4% (59/118) were female, and the mean age was 59.6 years (SD: 17.8) (Table 1). A history of extrapulmonary malignancy was present in 33.9% (40/118) of patients, while 8.5% (10/118) had a history of lung cancer. The primary indication for the procedure was initial diagnosis in 82.2% (97/118) of cases. Mediastinal lymph node biopsy was performed in 79.7% (94/118) of procedures. A definitive diagnosis was obtained in 92.4% (109/118) of cases.

**Table 1:** General characteristics SD, standard deviation; ASA, American Society of Anesthesiologists

Total population, n (%)	118 (100)
Female, n (%)	59 (50.4)
Age years, mean (SD)	59.56 (17.8)
History of non-pulmonary cancer, n (%)	40 (33.9)
History of pulmonary cancer, n (%)	10 (8.5)
Indication for procedure, n (%)	
Initial diagnosis	97 (82.2)
Station	16 (13.6)
Re-staging	5 (4.2)
Mediastinal nodule biopsy, n (%)	94 (79.7)
Pulmonary nodule or mass biopsy, n (%)	24 (20.3)
ASA score, n (%)	
1	13 (11)
2	53 (44.9)
3	45 (38.1)
4	7 (5.9)
Stations punctured, mean (SD)	1.36 (0.5)
Station punctured, n (%)	
11L	11 (9.3)
10L	11 (9.3)
7	84 (71.2)
4R	37 (31.4)
10R	24 (20.3)
Number of needle passes, mean (SD)	3.97 (1.3)
Number of cryogenic biopsies, mean (SD)	3.56 (1.1)
Freezing time min, mean (SD)	3.29 (0.5)
Nodule size mm, mean (SD)	23.53 (8.4)
Ultrasound characteristics of nodules, n (%)	
Elongated oval	49 (41.5)
Rounded	68 (57.6)
Distinct margins	90 (76.3)
Heterogeneous echogenicity	78 (66.1)
Calcifications	41 (34.7)
Central necrosis	85 (72)
Absence of hilum	77 (65.3)

Patients without a definitive diagnosis were older than those in the diagnostic group (67.0 years [SD: 18.7] vs. 58.9 years [SD: 17.7]) and more frequently had a history of extrapulmonary malignancy (66.7% [6/9] vs. 31.2% [34/109]) (Table 2).

**Table 2:** Clinical and procedural characteristics according to the diagnosis obtained SD, standard deviation; ASA, American Society of Anesthesiologists

	No diagnostics, n=9	Diagnostics, n=109
Female, n (%)	5 (55.6)	54 (50)
Age years, mean (SD)	67 (18.7)	58.94 (17.7)
History of non-pulmonary cancer, n (%)	6 (66.7)	34 (31.2)
History of pulmonary cancer, n (%)	0 (0)	10 (9.2)
Indication for procedure, n (%)		
Initial diagnosis	9 (100)	88 (80.7)
Station	0 (0)	16 (14.7)
Re-staging	0 (0)	5 (4.6)
Mediastinal nodule biopsy, n (%)	7 (77.8)	87 (79.8)
Pulmonary nodule or mass biopsy, n (%)	2 (22.2)	22 (20.2)
ASA score, n (%)		
1	2 (22.2)	11 (10.1)
2	3 (33.3)	50 (45.9)
3	3 (33.3)	42 (38.5)
4	1 (11.1)	6 (5.5)
Stations punctured, mean (SD)	1.22 (0.4)	1.38 (0.5)
Mediastinal lymph node stations, n (%)		
11L	1 (11.1)	10 (9.2)
10L	0 (0)	11 (10.1)
7	6 (66.7)	78 (71.6)
4R	1 (11.1)	36 (33)
10R	1 (11.1)	23 (21.1)
Number of needle passes, mean (SD)	3.67 (1.1)	4 (1.3)
Number of cryogenic biopsies, mean (SD)	3.33 (0.7)	3.58 (1.1)
Freezing time min, mean (SD)	3.33 (0.5)	3.28 (0.5)
Nodule size mm, mean (SD)	21.22 (10.5)	23.72 (8.2)
Ultrasound characteristics of nodules, n (%)		
Elongated oval	4 (44.4)	45 (41.3)
Rounded	4 (44.4)	64 (58.7)
Distinct margins	7 (77.8)	83 (76.1)
Heterogeneous echogenicity	6 (66.7)	72 (66.1)
Calcifications	4 (44.4)	37 (33.9)
Central necrosis	7 (77.8)	78 (71.6)
Absence of hilum	3 (33.3)	74 (67.9)
Punctured stations, mean (SD)	1.22 (0.4)	1.38 (0.5)

An average of 1.36 lymph node stations (SD: 0.5) were sampled per procedure. Mediastinal lymph node station 7 was the most frequently biopsied, accounting for 71.2% (84/118) of cases. The mean number of needle passes was 3.97 (SD: 1.3), and the average lymph node size was 23.5 mm (SD: 8.4). Regarding ultrasonographic characteristics, central necrosis was observed in 72.0% (85/118) of cases, well-defined margins in 76.3% (90/118), heterogeneous echogenicity in 66.1% (78/118), and absence of a visible hilar structure in 65.3% (77/118).

Patients with a successful diagnosis showed slightly higher mean numbers of sampled stations (1.38 [SD: 0.5] vs. 1.22 [SD: 0.4]), needle passes (4.0 [SD: 1.3] vs. 3.67 [SD: 1.1]), and cryobiopsy samples obtained (3.58 [SD: 1.1] vs. 3.33 [SD: 0.7]) compared with the non-diagnostic group (Table 2). Mean lymph node size was 23.7 mm (SD: 8.2) in the diagnostic group and 21.2 mm (SD: 10.5) in the non-diagnostic group. The absence of a visible hilar structure was more frequent in diagnostic procedures, occurring in 67.9% (74/109) compared with 33.3% (3/9) of non-diagnostic procedures.

The most frequent complications were pneumomediastinum in 11.9% (14/118) of patients, bronchospasm in 5.9% (7/118), and pneumothorax in 4.2% (5/118) (Table 3). Pneumomediastinum occurred in 12.8% (14/109) of patients with a definitive diagnosis and in none of the non-diagnostic cases (Table 4). Similarly, pneumothorax was observed in 4.6% (5/109) of diagnostic procedures. Bronchospasm occurred in 5.5% (6/109) of patients with a definitive diagnosis and in 11.1% (1/9) of those without a diagnosis.

**Table 3:** Histological results and safety TB, tuberculosis; PET-CT, positron emission tomography-computed tomography; PD-L1, programmed death-ligand 1; KRAS, Kirsten rat sarcoma viral oncogene homolog; ALK, anaplastic lymphoma kinase; BRAF, B-Raf proto-oncogene serine/threonine kinase; EGFR, epidermal growth factor receptor; spp., species

Total population, n (%)	118 (100)
Complications	
Folch score, n (%)	
1	1 (0.8)
2	104 (88.1)
3	12 (10.2)
4	1 (0.8)
Pneumomediastinum, n (%)	14 (11.9)
Pneumothorax, n (%)	5 (4.2)
Hemomediastinum, n (%)	0 (0)
Bronchospasm, n (%)	7 (5.9)
Infectious complication, n (%)	0 (0)
Histological diagnosis	
Fungal infection *Cryptococcus* spp, n (%)	4 (3.4)
Tuberculosis, n (%)	14 (11.9)
Non-TB mycobacteria, n (%)	1 (0.8)
Sarcoidosis, n (%)	5 (4.2)
Anthracosis, n (%)	17 (14.4)
Lymphoid hyperplasia, n (%)	14 (11.9)
Cancer, n (%)	
Squamous cell carcinoma of the lung	15 (12.7)
Lung adenocarcinoma	13 (11)
Small cell lung carcinoma	4 (3.4)
Lymphoma	33 (27.9)
Extracardiac metastasis	23 (19.4)
PET-CT, n (%)	24 (20.3)
Enhancing lung lesion, n (%)	5 (4.2)
Enhancing lymph nodes, n (%)	23 (19.5)
Number of lymph nodes showing uptake, n (%)	0.28 (0.6)
Molecular studies, n (%)	
PD-L1	44 (37.3)
KRAS	8 (6.8)
ALK	9 (7.6)
BRAF	7 (5.9)
EGFR	12 (10.2)

**Table 4:** Complications according to the diagnosis obtained

	No diagnostics, n=9	Diagnostics, n=109
Folch scale, n (%)		
1	0 (0)	1 (0.9)
2	8 (88.9)	96 (88.1)
3	1 (11.1)	11 (10.1)
4	0 (0)	1 (0.9)
Pneumomediastinum, n (%)	0 (0)	14 (12.8)
Pneumothorax, n (%)	0 (0)	5 (4.6)
Hemomediastinum, n (%)	0 (0)	0 (0)
Bronchospasm, n (%)	1 (11.1)	6 (5.5)
Infectious complication, n (%)	0 (0)	0 (0)

Anthracosis was the most common benign diagnosis, identified in 14.4% (17/118) of patients, followed by tuberculosis and lymphoid hyperplasia, each present in 11.9% (14/118) (Table 3). Among neoplastic diseases, lymphoma was the most frequent diagnosis, occurring in 27.9% (33/118) of patients, followed by extrathoracic metastases in 19.4% (23/118) and pulmonary squamous cell carcinoma in 12.7% (15/118).

PET-CT was performed in 20.3% (24/118) of patients. Hypermetabolic lymph nodes were identified in 19.5% (23/118), while hypermetabolic pulmonary lesions were detected in 4.2% (5/118). The mean number of hypermetabolic lymph nodes was 0.28 (SD: 0.6). Among molecular analyses, PD-L1 testing was the most frequently performed, being conducted in 37.3% (44/118) of patients, followed by EGFR testing in 10.2% (12/118).

## Discussion

This observational study describes the clinical and procedural characteristics of patients undergoing EBUS-TBNC. A definitive histopathological diagnosis was obtained in 92.4% of procedures. The technique also demonstrated a favorable safety profile, with predominantly mild complications and no severe adverse events such as hemomediastinum or infectious complications. Most procedures were performed for initial diagnostic purposes and involved mediastinal lymph nodes, with station 7 being the most frequently sampled site. Among histopathological findings, lymphoma was the most common neoplastic diagnosis, whereas anthracosis was the most frequent benign condition. In addition, tissue acquisition allowed molecular testing to be performed in a substantial proportion of patients.

A definitive diagnosis was achieved in 92.4% of patients undergoing the procedure, which is consistent with recent evidence supporting the utility of EBUS-TBNC [7,14,15]. A systematic review and meta-analysis by Mathew et al. [7] reported a pooled diagnostic yield of 91% for mediastinal cryobiopsy, exceeding that observed with conventional EBUS-TBNA (81%), particularly in benign diseases and lymphoproliferative disorders. Similarly, Chandragiri et al. [14] reported a pooled diagnostic yield of 92% for mediastinal cryobiopsy, highlighting its ability to obtain larger tissue samples with superior architectural preservation. These findings support the role of EBUS-TBNC as either a complementary or potentially superior technique to conventional needle aspiration.

The procedure demonstrated a favorable safety profile, characterized by the absence of major complications, hemomediastinum, or infectious events. These findings are consistent with recent meta-analyses reporting low complication rates associated with mediastinal cryobiopsy that are comparable to those observed with conventional EBUS-TBNA [7,16,17]. Mathew et al. [7] and Todisco et al. [16] reported that mediastinal cryobiopsy has a safety profile similar to that of needle aspiration, with infrequent clinically significant bleeding or adverse events requiring additional intervention. These characteristics support the implementation of EBUS-TBNC in centers with expertise in interventional bronchoscopy.

Most procedures were performed for initial diagnostic purposes and involved mediastinal lymph nodes, with station 7 being the most frequently biopsied site. This finding is expected, as the subcarinal station is one of the most accessible locations by EBUS and is commonly involved in both neoplastic and granulomatous diseases [17-20]. International EBUS-TBNA guidelines have consistently identified stations 4R, 4L, and 7 as the most frequently evaluated nodal stations during mediastinal staging and diagnostic assessment of mediastinal lymphadenopathy because of their accessibility and high diagnostic yield [17-20].

Among histopathological diagnoses, lymphoma was the most frequent neoplastic disease. The diagnosis of lymphoproliferative disorders may be limited by conventional EBUS-TBNA because of tissue fragmentation and loss of nodal architecture [20,21]. Recent studies have shown that mediastinal cryobiopsy significantly improves the acquisition of adequate tissue for the diagnosis and subclassification of lymphomas [22,23]. Poletti et al. [24] reported that EBUS-guided transbronchial mediastinal cryobiopsy may substantially contribute to the accurate diagnosis of lymphomas and uncommon mediastinal tumors while reducing the need for additional surgical procedures. Likewise, Mathew et al. [7] documented a significant increase in diagnostic yield for lymphoproliferative disorders when mediastinal cryobiopsy was employed.

The ability to perform molecular analyses in a considerable proportion of patients represents an important potential advantage of mediastinal cryobiopsy in the era of precision medicine [25]. In our cohort, molecular testing was performed in a substantial proportion of patients, with PD-L1 being the most frequently assessed biomarker (37.3%), followed by EGFR (10.2%), ALK (7.6%), KRAS (6.8%), and BRAF (5.9%). The relatively frequent use of molecular testing may reflect the ability of cryobiopsy to obtain larger and more cellular tissue specimens, which can provide greater material for immunohistochemistry and genomic analyses [13,25,26]. Recent studies have similarly reported higher tumor cellularity and improved tissue preservation in cryobiopsy specimens, facilitating molecular characterization and potentially reducing the need for repeat procedures [25,26]. Soo et al. [26] reported higher success rates for detecting molecular alterations using next-generation sequencing in cryobiopsy samples, supporting the potential role of this technique in optimizing tissue acquisition for targeted therapies and immunotherapy.

Limitations

This study has several limitations. First, its observational design is subject to potential selection and information biases inherent to the use of clinical records and retrospective data collection. Second, because this was a descriptive study without a comparator group, causal associations could not be established, and no multivariable analysis was performed to identify independent factors associated with diagnostic success or complications. Third, systematic follow-up of non-diagnostic cases was not available, precluding confirmation of potential false-negative results and potentially influencing the reported diagnostic yield. Fourth, the single-center nature of the study may limit the external validity and generalizability of the findings to other clinical settings. Finally, the relatively small sample size, particularly within the non-diagnostic group, may have reduced the precision of some estimates. Prospective multicenter comparative studies are warranted to validate these findings and further define the role of EBUS-TBNC in clinical practice.

## Conclusions

EBUS-TBNC demonstrated a high rate of definitive diagnosis in this real-world cohort of patients undergoing evaluation of mediastinal lymphadenopathy and pulmonary lesions, with a diagnosis obtained in 92.4% of cases. The technique provided tissue suitable for the diagnosis of a broad spectrum of malignant and benign conditions, including lymphoma, metastatic disease, primary lung cancer, tuberculosis, sarcoidosis, and reactive lymphoid processes. The safety profile was generally acceptable. Most bleeding events were low grade according to the Folch classification, while pneumomediastinum, bronchospasm, and pneumothorax were the most frequently observed complications. No hemomediastinum or infectious complications were identified. The availability of specimens for PD-L1 assessment and molecular testing for EGFR, ALK, KRAS, and BRAF further highlights the potential utility of cryobiopsy in contemporary diagnostic pathways, particularly when tissue is required for both histopathological and molecular characterization.

Overall, EBUS-TBNC appears to be a feasible and potentially valuable complementary technique for obtaining diagnostic tissue from mediastinal and pulmonary lesions, combining a high observed diagnostic yield with an acceptable safety profile. However, the retrospective single-center design, limited sample size, absence of a direct comparator, and lack of formal diagnostic accuracy assessment limit the generalizability of these findings.
